# DNA-Binding and Topoisomerase-I-Suppressing Activities of Novel Vanadium Compound Van-7

**DOI:** 10.1155/2012/756374

**Published:** 2012-09-30

**Authors:** Xiao-mei Mo, Zhan-fang Chen, Xin Qi, Yan-tuan Li, Jing Li

**Affiliations:** ^1^Key Laboratory of Marine Drugs, Ministry of Education, School of Medicine and Pharmacy, Ocean University of China, 5 Yu-Shan Road, Qingdao 266003, China; ^2^The Department of Pharmacy, Qingdao Women and Children Hospital, 127 Liao-Yang West Road, Qingdao 266034, China

## Abstract

Vanadium compounds were studied during recent years to be considered as a representative of a new class of nonplatinum metal anticancer agents in combination to its low toxicity. Here, we found a vanadium compound Van-7 as an inhibitor of Topo I other than Topo II using topoisomerase-mediated supercoiled DNA relaxation assay. Agarose gel electrophoresis and comet assay showed that Van-7 treatment did not produce cleavable complexes like HCPT, thereby suggesting that Topo I inhibition occurred upstream of the relegation step. Further studies revealed that Van-7 inhibited Topo I DNA binding involved in its intercalating DNA. Van-7 did not affect the catalytic activity of DNase I even up to100 **μ**M. Van-7 significantly suppressed the growth of cancer cell lines with IC_50_ at nanomolar concentrations and arrested cell cycle of A549 cells at G2/M phase. All these results indicate that Van-7 is a potential selective Topo I inhibitor with anticancer activities as a kind of Topo I suppressor, not Topo I poison.

## 1. Introduction

DNA topoisomerases which catalyze the interconversions of various topological states of DNA were originally discovered as activities that change the superhelical structure of closed circular DNAs [1]. Based on their functional mechanisms, DNA topoisomerases have been classified into two types: type I DNA topoisomerases (Topo I) break and rejoin only one of the two strands during catalysis, while type II DNA topoisomerases (Topo II) break and rejoin both strands for each DNA strand-passing reaction. Studies have shown that Topo I is associated with actively transcribed genes, whereas Topo II is required for DNA replication and for successful traverse of mitosis [2, 3]. Thus DNA topoisomerases modify the topological states of DNA which facilitate various DNA transactions such as DNA replication, recombination, chromosome condensation/decondensation, and chromosome segregation. Previous studies have suggested that Topo I does not require a nucleotide cofactor or any other energy source to relax supercoiled DNA while Topo II cannot relax supercoiled DNA without ATP [4].

 Studies have identified DNA topoisomerases as therapeutic targets in cancer chemotherapy [5]. Topo I is a molecular target of hydroxycamptothecine (HCPT) while Topo II is a molecular target of a number of clinically useful anticancer drugs such as etoposide (VP-16), doxorubicin, mitoxantrone and (N-[4-(9-acridinylamino)-3-methoxyphenyl] methanesulphonanilide) (m-AMSA). Other compounds such as saintopin, intoplicine, indoloquinolinedione derivatives, *β*-lapachone, and related naphthoquinones have been shown to act on both Topo I and Topo II [6–9]. 

**Figure 1:**
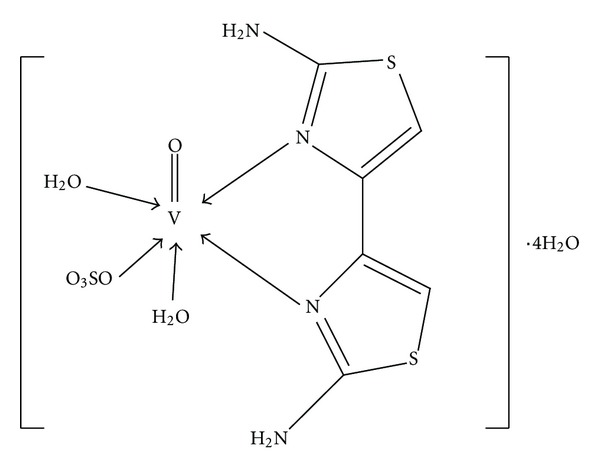
Van-7, [VO(SO_4_)(C_6_H_6_N_4_S_2_)(H_2_O)_2_]4H_2_O.

 The success of platinum as anticancer agent has stimulated a search for other metallic cytotoxic compounds with equal or greater anticancer activity and lower toxicity [10]. Three platinum-based antineoplastic agents are now in routine clinical practice: cisplatin, carboplatin, and oxaliplatin [11]. Although these heavy metal agents are active against a variety of cancers, their clinical applications are associated with severe side effects including gastrointestinal symptoms (nausea, vomiting, diarrhea, and abdominal pain), renal tubular injury, neuron-muscular complications, and ototoxicity. In addition, the use of platinum is limited in many tumor types by primary and acquired resistance to this agent [12]. This has led to an ongoing quest for the discovery of nonplatinum metals that may extend the spectrum of activity of metal-based drugs [13]. Vanadium compounds have been widely reported to exert preventive effects against chemical carcinogenesis on animals, by modifying various xenobiotic enzymes and inhibiting carcinogen-derived active metabolites [14, 15]. In the present paper, we investigated the effects of a new vanadium compound, diaqua (2,2′-diamino-4,4′-bi-1,3-thiazole) oxosulfato-vanadium (IV) tetrahydrate (Van-7, Figure 1) [16], on the capability of inhibiting Topo I and anticancer activities in vitro. We find that the Van-7 is a potential Topo I inhibitor as a kind of Topo I suppressor other than Topo I poison.

## 2. Materials and Methods

### 2.1. Drugs and Reagents

Van-7 in light blue color crystalloid was provided by the Pharmacochemistry Department III of Marine and Food institute, Ocean University of China and diluted in double-distilled water. The purity was determined by RP-HPLC to be more than 99.0%. Anal. Calcd for VC_6_H_18_N_4_O_11_S_3_ (M.W. 469.36): C, 15.35; H, 3.86; N, 11.94%. Found: C, 15.30; H, 3.81; N, 11.92%. Supercoiled plasmid pBR322 was purchased from Takara Biotechnology Company (Dalian, China). 3-[4,5-dimethylthiazol-2-yl]-2,5-diphenyltetrazolium bromide (MTT), Proteinase K, and SDS were purchased from Sigma. VP16 was obtained from Pudong Pharmaceutical Factory (China) and HCPT from Feiyun Pharmaceutical Factory (China). Other chemicals used were all of analytical reagent grade. Human DNA Topo I was gifted by ZHOU Shi-Ning (Sun Yat-Sen University, China) [17].

### 2.2. Topo II Preparation

Topo II was extracted from L-1210 leukemic cells in peritoneal cavity 7 days after tumor inoculation following the procedure of De Isabella et al. [12, 18]. In brief, harvested L-1210 cells from DBA/2 mouse were resuspended in 10 mL of buffer A (10 mM Tris (pH 7.5), 1.5 mM MgCl_2_, and 10 mM NaCl) and allowed to sit at 0°C for 10 min. A nonionic detergent (1 mL of 10% Nonidet P-40) was added, and the mixture gently triturated and finally left at 0°C for 15 min. The cells were then homogenized and centrifuged at 2500 rpm for 10 min, and the pellet was resuspended in 2 mL of buffer B (50 mM Tris (pH 7.5), 25 mM KCl, 2 mM CaCl_2_, 3 mM MgCl_2_, and 0.25 M sucrose). The nuclei thus obtained were layered over 0.6 mL of buffer C (buffer B with 0.6 M sucrose) and sedimented at 7000 rpm for 10 min. The pellet was resuspended in 2 mL of buffer D (buffer B without CaCl_2_, and with 5 mM MgCl_2_), centrifuged at 7000 rpm for 10 min, and finally resuspended in 0.3 mL of buffer E (same as buffer D without sucrose). The solution was added 30 *μ*L of 0.2 M EDTA (pH 8.0) and 0.66 mL of buffer F (80 mM Tris (pH 7.5), 2 mM EDTA, 1 mM DTT, 0.53 M NaCl, and 20% glycerol (v/v)). This mixture was gently triturated, left at 0°C for 30 min, and centrifuged at 40000 rpm for 20 min. The supernatant from the last centrifugation contains Topo II activity, which was examined by the DNA relaxation assay. One unit of Topo II was defined as the amount of enzyme required to fully relax 0.5 *μ*g of supercoiled DNA under the conditions described below. 

### 2.3. DNA Relaxation Assay

Topoisomerases were assayed by relaxation of supercoiled plasmid DNA [19, 20]. Relaxation of 250 ng of supercoiled by Topo I (2 U) was performed in 20 *μ*L of Topo I relaxation buffer (10 mM tris-HCl, pH 7.9, 1 mM EDTA, 150 mM NaCl, 0.1% (w/v) BSA, 0.1 mM spermidine, 5% (v/v) glycerol), in the presence and absence of varying amounts of the test compounds, dissolved in dimethyl sulfoxide (5% (v/v) final concentration). Reactions were started by the addition of DNA. Control groups were either DNA alone or DNA treated with topoisomerase. Relaxation of supercoiled DNA with Topo II was performed in Topo II relaxation buffer (50 mM Tris-HCl, pH 8.0, 0.5 mM ATP, 10 mM MgCl_2_, 120 mM NaCl, and 0.5 mM dithiothreitol). DNA was added before the addition of Topo II. After 30 min at 37°C, the reaction was terminated by the addition of 1% (w/v) SDS and digested with 50 mg/mL proteinase K at 55°C for 30 min. DNA was extracted with an equal volume of chloroform/isoamyl alcohol (24 : 1) and separated on 1% (w/v) agarose gel in Tris-acetate-EDTA (TAE) buffer (40 mM trisacetate, pH 8.0, and 2 mM EDTA) at 2 V/cm for 3.5 h. Gels were stained with 5 mg/mL ethidium bromide, destained, and photographed using Polaroid 665 film or a gel-imaging system for numerical quantification by densitometry scanning (Herolab, Wiesloch, Germany). 

### 2.4. Comet Assay

Nuclei were isolated from P388 cells by incubating whole cells in nuclear buffer (5 mM MgCl_2_, 1 mM EGTA, 1 mM KH_2_PO_4_, 150 mM NaCl) for 20 min on ice with gentle rocking. Plasma membrane disruption and nuclei integrity was checked under the microscope. Isolated nuclei were exposed to Van-7 or HCPT at 40 *μ*M for 30 min at 37°C. DNA break was detected as previously described [21]. Briefly, nuclei were embedded in agarose gel and then spread on a polylysinated microscope slide. Nuclei were lysed in lysis buffer (2.5 M NaCl, 10 mM Tris-HCl, 100 mM Na_2_EDTA, 1% Triton, 10% DMSO, pH 10) for 1 h at 4°C. After lysis, nuclei were preincubated for 20 min at 4°C in the electrophoresis buffer (0.3 M NaOH, 1 mM Na_2_EDTA, pH 13.5) and then subjected to alkaline gel electrophoresis (300 mA, 4°C, 20 min). Slides were analysed by laser scanning microscopes (LSM, Zeiss Ltd.) to quantitative DNA damage. The tail moment, calculated with Komet 5.5 software (Kinetic Imaging, Bath, UK) by multiplying the total intensity of the comet tail by the migration distance from the center of the comet head, was used to measure DNA damage. Fifty nuclei for each experimental point were scored blind from two slides. The frequency distribution was defined as the percentage of number of cells with tail moment value in total cells scored.

### 2.5. Measurement of Topo-I Mediated DNA Cleavage

Reaction mixtures containing pBR322 DNA (250 ng) and excess of enzymes (i.e., 100 U of Topo-I) and drugs were incubated at 37°C for 30 min. Samples, which contained tested drugs, were assembled in this order: DNA, Topo-I, Van-7, or HCPT. The reactions were terminated by the adding 1% SDS and 150 mg/mL proteinase K. After the additional 30 min incubation at 37°, DNA samples were electrophoresed in 1% agarose gel containing 0.5 mg/mL ethidium bromide.

### 2.6. Analysis of Topo I/DNA Binding by EMSA

EMSA (electrophoretic mobility shift assay) was basically performed as described elsewhere [22]. In brief, 250 ng of supercoiled pBR322 DNA was incubated in 20 *μ*L of relaxation Topo I buffer with or without excess of Topo I (100 U) in the presence of the test compounds at 37°C for 6 min. The reaction was started by the addition of DNA. The samples containing test compounds were assembled in the order of Topo I, HCPT, or Van-7. Samples were immediately loaded onto the 0.8% agarose gel in Tris-acetate-EDTA buffer with 1 *μ*g/mL ethidium bromide and separated by electrophoresis for 6 h at 2 V/cm. 

### 2.7. Ethidium Bromide Displacement Fluorescence Assay

Ethidium bromide displacement fluorescence assay [23] was employed to determine whether Van-7 binds to DNA. Fluorescence emission spectra (*λ*_max⁡_ = 600 nm, excitation wavelength 546 nm) were obtained at 25°C on a Beckman fluorescence spectrophotometer. The assays contained 1 *μ*M ethidium bromide, 0–100 *μ*M Van-7, and 1 *μ*g supercoiled pBR322 DNA in 2 mL of fluorescence buffer. 

### 2.8. Measurement of DNase I Activity

Bovine DNase I (4.0 U/mL) was incubated with 400 ng of pBR322 DNA in 20 *μ*L of buffer (50 mM Tris-HCl, pH 7.5, 10 mM MnCl_2_, and 50 *μ*g/mL BSA) in the presence of Van-7 (50–100 *μ*M) for 15 min at 37°C. The reaction was stopped by the addition of 25 mM EDTA (final concentration) and followed by agarose gel electrophoresis.

### 2.9. MTT Assay

A549, Hela, BEL-7402, P388, and L-02 cells were purchased from American Type Culture Collection (ATCC). Culture media were selected according to ATCC suggestions. To perform growth experiments cells were seeded (10,000 cells/well) in 96-well flat bottom plates. After 24 h the media were replaced, and, after one washing, media containing the drugs were added. After 48 h incubation at 37°C, MTT solution was added at 5 mg/mL and incubated for an additional 4 h. Then culture supernate was removed, and 150 *μ*L dimethyl sulfoxide (DMSO) was added per well to dissolve the formazan crystals. Colorimetric determination was made at 570 nm using a microplate reader (Spectra Rainbow, Austria). Six parallel samples were prepared in each group, and each experiment has been replicated for three times. A dose-response study was performed to calculate the 50% inhibiting concentration (IC_50_) for Van-7. IC_50_ calculated by the application of the Reed and Muench method [24] is as follows:

(1) $$\begin{array}{c} {\text{IC}}_{50}=\text{antilog}\left\{A+\left[\left(\frac{B}{C}\right)D\right]\right\}, \end{array}$$

where *A *is log concentration below 50% mortality, *B *is 50 − mortality below 50%, *C *is mortality above 50%  − mortality below 50%, and *D *is log concentration above 50%  − log concentration below 50%.

### 2.10. Flow Cytometry

A549 human lung cancer cells were seeded (300,000 cells/well) in 6-well flat bottom plates. After an incubation in F-12 medium containing 10% FCS (v/v) at 37°C for 24 h, Van-7 was added at 100 *μ*M, 50 *μ*M, 25 *μ*M, 12.5 *μ*M (final concentration), and HCPT at 40 *μ*M except the blank control group. 48 h later, A549 cells were harvested, washed three times with phosphate-buffered saline (PBS), stained with PI for 30 min, gated and analyzed by FCM (Becton and Dickinson, Vantage, USA) with a 488 nm laser excitation and a 530 nm emission filter. Data were analyzed with Modfit 2.0 and two parallel samples were prepared in each group, and each experiment was replicated for three times.

### 2.11. Statistical Analysis

Comparisons of treatment outcomes were tested for statistically significant differences using Student's *t*-test for paired data. Statistical significance was assumed at *P ≤ 0.05, **P ≤ 0.01, and ***P ≤ 0.001.

## 3. Results

### 3.1. Inhibition of the Activity of Topo I but Not Topo II by Van-7

**Figure 2:**
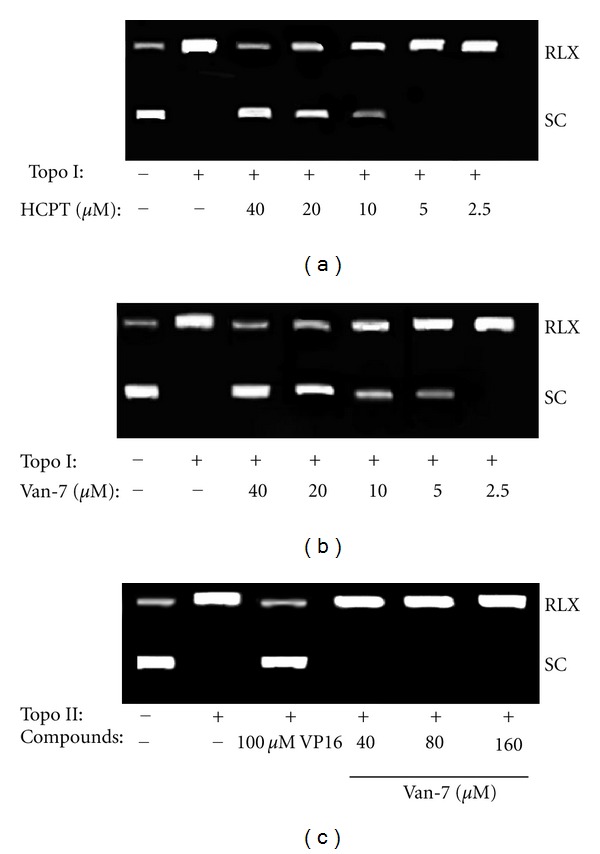
(a) Effect of HCPT on Topo I-mediated supercoiled pBR322 relaxation. (b) Effect of Van-7 on Topo I-mediated supercoiled pBR322 relaxation. Both of the two reactions were carried out without ATP. (c) Effect of Van-7 on Topo II-mediated supercoiled pBR322 relaxation. The reaction was carried out in presence of ATP. RLX: relaxed DNA, a circular DNA molecule that has no superhelical turns. SC: supercoiled DNA,over- or underwinding of a DNA strand.

The effects of Van-7 on topoisomerases were investigated using a conventional plasmid DNA relaxation assay. HCPT, a well-known Topo I inhibitor, was employed as a positive control. A representative experiment is shown in Figure 2, the inhibition of the DNA relaxation activity of Topo I by HCPT or Van-7 was in concentration-dependent manner. We found that Van-7 at the concentration of 5 *μ*M obviously inhibited the DNA relaxation activity of Topo I, while HCPT did not have such effect as no visible electrophoresis band of supercoiled DNA was displayed at 5 *μ*M. In additional, Van-7 also completely inhibited the DNA relaxation activity of Topo I at the concentration of 40 *μ*M. These results suggest that Van-7 is a more potent inhibitor of Topo I than HCPT.

To investigate if Van-7 is a selective inhibitor of Topo I, we further tested its effect on the catalytic activity of Topo II. As shown in Figure 2(c), no inhibitory activity was observed against Topo II, even up to the concentration of 160 *μ*M. On the contrary, VP16, a well-known Topo II inhibitor, obviously inhibited the strand passage activity of Topo II at 100 *μ*M. 

### 3.2. Van-7 Does Not Induce Cleavable Complex Formation Like HCPT

**Figure 3:**
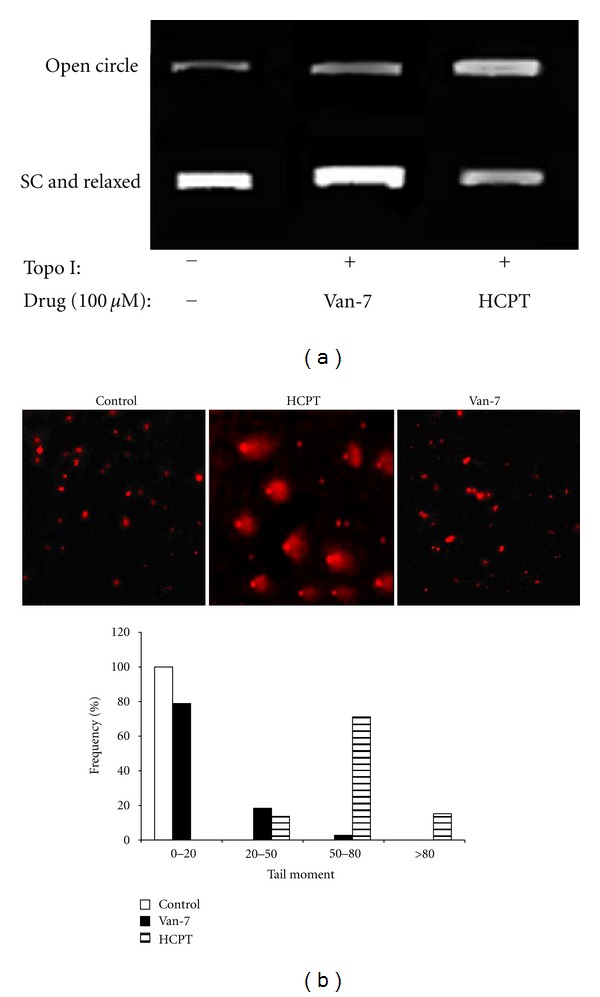
(a) Representative image of agarose gel electrophoresis of the cleavage assay. Only HCPT induced the formation of a slow migrating complex formed by enzyme, drug, and DNA, whereas Van-7 did not. (b) Comet assay performed in isolated nuclei in the presence of the vehicle, HCPT (40 *μ*M), or Van-7 (40 *μ*M). Tail moment for each comet was calculated. Nuclei were grouped according to the tail moment value in three groups, and the frequency distribution is shown in the bar chart. DNA damage is increased in nuclei with the highest tail moment values.

Topo I acted as a single-strand endonuclease and ligase, and HCPT inhibits ligase without affecting the cleavage step. Therefore, HCPT entraps a slow migrating complex formed by the enzyme, the drug, and DNA, named as cleavable complex [25]. We further assessed whether Van-7 also induced the formation of the cleavable complexes. As shown in Figure 3(a), obvious increase of electrophoresis band for open circle was observed after HCPT treatment, suggesting that HCPT could induce formation of cleavable complex; however, Van-7 was not similar to HCPT as no obvious cleavable complex was found comparing with control group, suggesting that inhibitory mechanism of Van-7 against Topo I is different from that of HCPT.

To further consolidate the presumption above, the freshly isolated cell nuclei were treated for 30 min with the compound, and the occurrence of DNA breaks was assessed by comet assay (Figure 3(b)). By comet assays, HCPT was found to be able to induce DNA breaks with obvious formation of comet tail, whereas Van-7 was unable to produce a similar effect.

### 3.3. Van-7 Exhibits Notable DNA-Binding Activity via the Intercalative Mode

**Figure 4:**
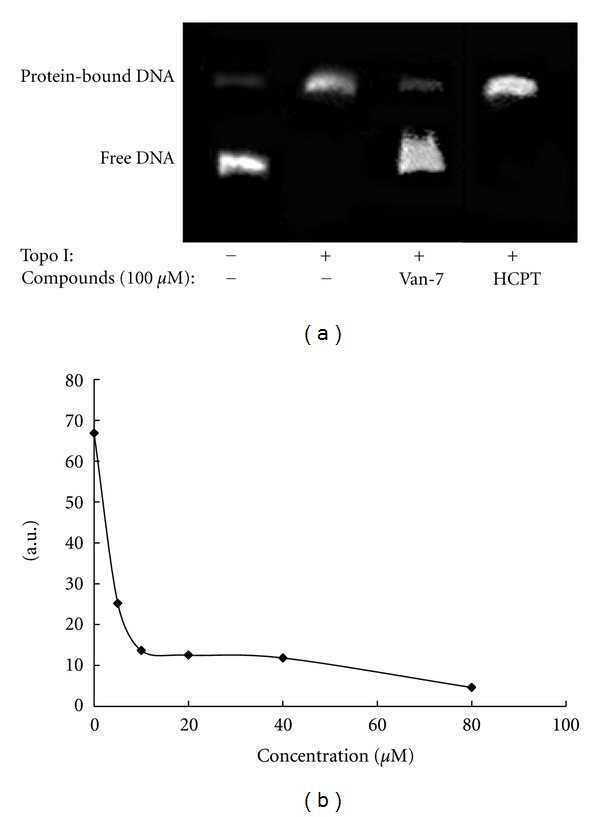
(a) Representative image of the agarose gel electrophoresis for EMSA between pBR322 DNA and Topo I. HCPT did not modulate the formation of the complex DNA/Topo 1, whereas Van-7 significantly hampered the binding of the enzyme to DNA. (b) Van-7 displaces ethidium bromide from DNA strand. The ability of Van-7 to interact with DNA was determined by a fluorescence-based ethidium bromide displacement assay. Samples contained 1 *μ*M ethidium bromide and 1 *μ*g DNA pBR322. Increasing concentrations of Van-7 were added, and decreasing ethidium fluorescence at 600 nm (*λ*_max⁡_) was monitored (546 nm excitation wavelength).

 We next investigated whether Van-7 directly interfered with binding of Topo I to DNA using an EMSA assay. Here, HCPT was selected as a control because it can inhibit the ligase activity and does not interfere with the binding of the enzyme to DNA [26]. As shown in Figure 4(a), Van-7 at 100 *μ*M significantly hampered the binding of the enzyme to DNA, and this did not occur with HCPT as expected.

 Ethidium bromide is a large, flat basic molecule that resembles a DNA base pair. Because of its chemical structure, it can intercalate (or insert) into a DNA strand. Displacement of ethidium bromide from DNA with concomitant reduction in ethidium fluorescence was used as an approach to examine whether a compound could intercalate into DNA. As shown in Figure 4(b), with the increase of Van-7, marked reduction in fluorescence intensity was found accordingly, suggesting that ethidium bromide could be displaced by Van-7 from DNA strand. This result indicates that Van-7 can intercalate into DNA and bind to DNA.

### 3.4. Van-7 Does Not Affect the Activity of DNase I

**Figure 5:**
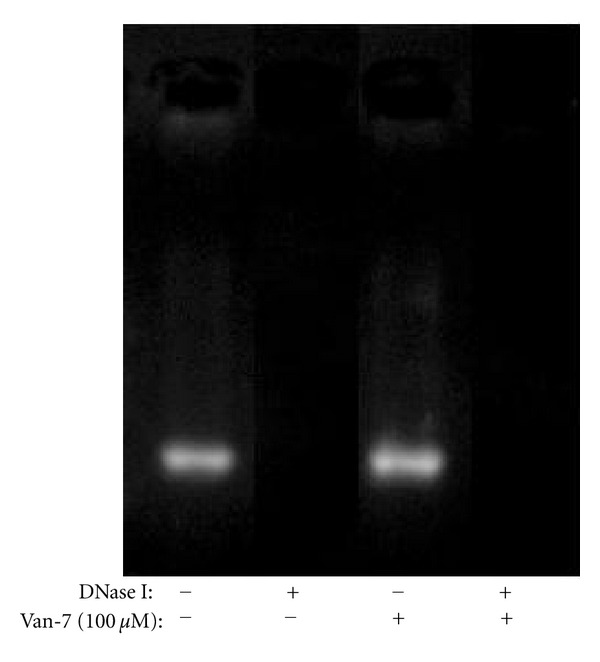
Representative image of agarose gel electrophoresis of the DNase assay. Bovine DNase I (4.0 U/mL) was incubated with 400 ng of pBR322 DNA in the presence of Van-7 (100 *μ*M) for 15 min at 37°C. Van-7 was unable to interfere with DNase activity. This experiment was repeated three times with similar results.

We further detected the effect of Van-7 on the catalytic activity of bovine DNase I. As shown in Figure 5, with Van-7 even up to100 *μ*M or without Van-7, the DNase I could digest DNA indistinguishably, suggesting that Van-7 does not affect the catalytic activity of DNase I. 

### 3.5. The Cytotoxicity Activities of Van-7 In Vitro

Van-7 was tested in four human cancer cell lines to observe its anticancer activities in vitro. Data were shown in Table 1; Van-7 was able to significantly inhibit the growth of the cancer cell lines, but show faint inhibition activity to human normal liver cell line L-02.

### 3.6. Van-7 Arrests A549 Cell Line at G2/M Phase

**Figure 6:**
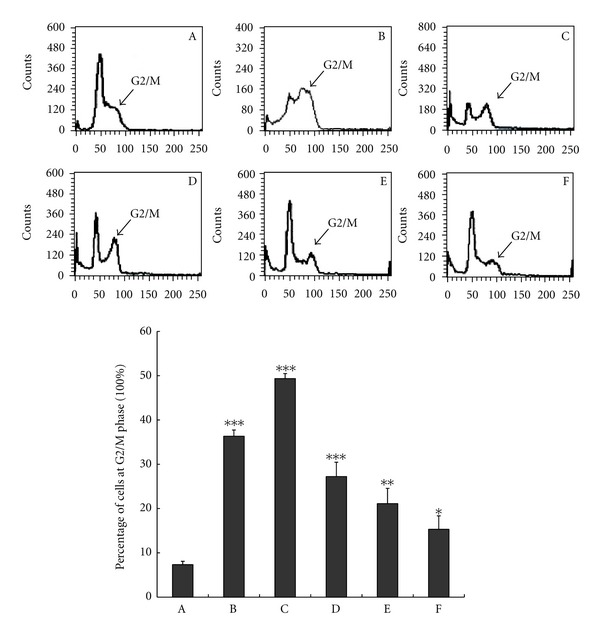
Van-7 arrested A549 cell line at G2/M phase. A: control; B: HCPT, 40 *μ*M; C: Van-7, 100 *μ*M; D: Van-7, 50 *μ*M; E: Van-7, 25 *μ*M; F: Van-7, 12.5 *μ*M. The percentage of G2/M, A: 7.32%; B: 36.32%; C: 49.36%; D: 27.21%; E: 21.10%; F: 15.31%. The values were the means ± SD, *n* = 3. *P < 0.05, **P < 0.01, ***P < 0.001 versus control group.

To clarify the pattern of Van-7-induced growth suppression, A549 cells were treated with HCPT or Van-7 for 48 hours and analyzed by flow cytometry. Flow cytometry analysis showed that Van-7 treatment resulted in the accumulation of cell populations in G2/M phase in a concentration-dependent manner (Figure 6), while HCPT blocked the cells at S phase [27]. Treatment of A549 cells with 100 *μ*M, 50 *μ*M, 25 *μ*M, and 12.5 *μ*M of Van-7 increased the percentage of cells in the G2/M phase to 49.36%, 27.21%, 21.10%, and 15.31%, respectively.

## 4. Discussion

Since the discovery of *cis*-platinum, many transition metal complexes have been synthesized and assayed for antineoplastic activity. In recent years, vanadium-based molecules have emerged as promising anticancer and antimetastatic agents with potential application in platinum-resistant tumors or as alternatives to platinum [14]. In our screening model for inhibitor of DNA topoisomerases, we discover that Van-7, one kind of vanadium compound, has a strongly inhibitory activity to Topo I, not to Topo II.

Topo I inhibitors include Topo I poison and Topo I suppressor. Both of them are agents designed to interfere with the action of topoisomerase enzymes, but the mechanisms are different. HCPT, as a kind of camptothecin derivative, is a typical Topo I poison. HCPT can stabilize DNA Topo I, forming drug DNA Topo I complex and inhibiting Topo I activity [28]. However, suppressor is DNA conjugant (such as Hoechst33258) or intercalator (such as aclacinomycin A). Topo I suppressor combines with DNA or deforms the structure of DNA to inhibit the catalytic activity of Topo I to result in cell death. To exert this effect, most Topo I suppressors must be in relatively high concentration, and the activity of DNA conjugant depends on its closely binding with DNA. In the present paper, Van-7 was found to inhibit the activity of Topo I obviously; however, in the test of drug DNA Topo I complex with gel electrophoresis analysis, Van-7 was not found to form cleavable complex even up to 100 *μ*M. In order to further evaluate Van-7 a direct involvement in the inhibition of topoisomerase in nucleus independently on some potential interference, freshly isolated cell nuclei were used, and the probable occurrence of DNA breaks was assessed by comet assay. We found that HCPT treatment showed obvious comet tail while Van-7 did not. From the above two results, we confirm that inhibitory effect of Van-7 on Topo I activity is different from that of HCPT, and the fact that Van-7 cannot form Drug-DNA Topo I complex indicates that Van-7 is not a poison. Therefore, we presume that Van-7 acts on the upstream of catalytic reaction and probably disturbs the combination of Topo I with DNA. 

DNA mobility shift assay (EMSA), also called gel retardation assay, can be used to detect DNA-protein interaction in vitro. In electric field, DNA fragments binding with protein migrate more slowly to positive pole than free DNA fragments. We further evaluate the effect of the compound on the binding of DNA with Topo I. In our tests, we found that Van-7 at 40 *μ*M obviously inhibited the binding of Topo I to DNA with free DNA fragments band in gel electrophoresis. But this phenomenon was not observed in the present of HCPT. In additional, Van-7 significantly reduced the fluorescence intensity by displaceing ethidium bromide from DNA strand. All these results indicate that Van-7 intercalates to DNA and exert its inhibitory effect to Topo I.

DNase I is a nuclease that cleaves DNA preferentially at phosphodiester linkages adjacent to a pyrimidine nucleotide, yielding 5′-phosphate-terminated polynucleotide with a free hydroxyl group on position 3′, which is similar to topoisomerases cutting the phosphate backbone of the DNA; however, Van-7 was not found to affect the activity of Dnase I, which indicates that Van-7 inhibits activity of Topo I with some selectivity.

Topo I has significant consequences for cancer and cancer chemotherapy via their antiproliferative or cell-differentiating action. From the results of MTT tests, Van-7 was found to strongly inhibit the growth of tumor cells, such as BEL-740A549 and Hela cells, but not normal cells. As the specific activity of Topo I was about 4-fold greater in proliferating (log phase) cells than in nonproliferating (confluent) cells, and in contrast to the changes in Topo I levels, the specific activity of Topo II showed no detectable difference in proliferating versus non-proliferating cells [29], therefore it is reasonable that Van-7 is more selective to cancer cells with proliferation rate much higher than normal cells. It is reported that the compounds exhibit a different inhibitory mechanism from camptothecin that may induce different phase cell cycle arrest, a novel Topo I inhibitor with repressing the catalytic cleavage activity of Topo I instead of forming the drug-enzyme-DNA covalent ternary complex arrested cell cycle at G2/M phase to K562 Cells [30]. Our FACS analysis result that Van-7 in the concentration of 100 *μ*M could block the cell cycle to G2-M other than S phase is consistent with this report.

On the whole, we consider that Van-7 is a potential Topo I inhibitor as a kind of Topo I suppressor. Van-7 can insert into DNA base pairs, resulting in DNA structure distortion, and then inhibiting the binding of DNA to Topo 1, finally affecting the catalytic activity of Topo I. To the best of our knowledge, it is the first report about clarifying inhibitory ability of a vanadium metal compounds on Topo-I catalytic activity and its mechanism. However, the anticancer activity in vivo and detail mechanisms of Van-7 requires further investigation.

## Figures and Tables

**Table 1:** IC_50_ (*μ*M) values obtained after 72 h of continuous Van-7 exposure.

Cell lines	P388	BEL-7402	A549	Hela	L-02
	0.9 ± 0.2	5.3 ± 1.1	0.1 ± 0.04	5.1 ± 1.2	60.2 ± 7.6

## Abbreviations

Topo I: Topoisomerase I
Topo II: Topoisomerase II
HCPT: Hydroxycamptothecine
DMSO: Dimethylsulfoxide
VP16: Etoposide.
